# Determinants of active aging and quality of life among older adults: systematic review

**DOI:** 10.3389/fpubh.2023.1193789

**Published:** 2023-06-26

**Authors:** Roy Rillera Marzo, Praval Khanal, Sunil Shrestha, Devi Mohan, Phyo K. Myint, Tin Tin Su

**Affiliations:** ^1^Global Public Health, Jeffrey Cheah School of Medicine & Health Sciences, Monash University Malaysia, Bandar Sunway, Selangor Darul Ehsan, Malaysia; ^2^Institute of Applied Health Sciences, School of Medicine, Medical Sciences & Nutrition, University of Aberdeen, Aberdeen, United Kingdom; ^3^Department of Community Medicine, International Medical School, Management and Science University, Selangor, Malaysia; ^4^Nepal Health Research and Innovation Foundation, Kathmandu, Nepal; ^5^School of Pharmacy, Monash University Malaysia, Bandar Sunway, Selangor Darul Ehsan, Malaysia; ^6^South East Asia Community Observatory (SEACO), Monash University Malaysia, Bandar Sunway, Selangor Darul Ehsan, Malaysia

**Keywords:** active aging, quality of life, older adults, health, participation, security

## Abstract

**Introduction:**

Population demography across the globe shows an increasing trend in the aging population due to better healthcare, improved nutrition, advanced health-related technology, and decreased fertility rate. Despite these advancements, there remains a knowledge gap in understanding the association between active aging determinants and quality of life (QoL) among older adults, particularly within diverse cultural contexts, which has not been adequately explored in previous research. Therefore, understanding the association between active aging determinants and QoL can help policymakers plan early interventions or programs to assist future older adults in both aging actively and optimizing their quality of life (QoL), as these two factors have a bidirectional relationship.

**Objective:**

This study aimed to review evidence regarding the association between active aging and quality of life (QoL) among older adults and to determine the most widely used study designs and measurement instruments in studies conducted between 2000 and 2020.

**Methods:**

Relevant studies were identified by a systematic search of four electronic databases and cross-reference lists. Original studies examining the association between active aging and QoL in individuals aged 60 years or older were considered. The quality of the included studies and the direction and consistency of the association between active aging and QoL were assessed.

**Results:**

A total of 26 studies met the inclusion criteria and were included in this systematic review. Most studies reported a positive association between active aging and QoL among older adults. Active aging had a consistent association with various QoL domains including physical environment, health and social services, social environment, economic, personal, and behavioral determinants.

**Conclusion:**

Active aging had a positive and consistent association with several QoL domains among older adults, backing the notion that the better the active aging determinants, the better the QoL among older adults. Considering the broader literature, it is necessary to facilitate and encourage the active participation of older adults in physical, social, and economic activities for the maintenance and/or improvement of QoL. Identifying other possible determinants and enhancing the methods to improve those determinants may help improve the QoL among older adults.

## Introduction

Population demography across the globe shows an increasing trend in the aging population due to better healthcare, improved nutrition, advanced health-related technology, and decreased fertility rate (1). By the year 2050, the global population of older adults is expected to increase by approximately 20.6%, resulting in an estimated 2 billion older adults worldwide. Most of these older adults will live in low- and middle-income countries [LMICs; (2)]. Due to this rapid demographic transition, there will be a potential shortage of the productive young population in the coming decades (2). Therefore, it is essential to develop strategies by which older people can be actively engaged to promote their wellbeing and that of their families. In contrast to previous studies in this area (3–5), which primarily focused on specific disease conditions or were conducted in developed regions, our study adopted a comprehensive approach to examine the association between active aging determinants and quality of life (QoL) among community-dwelling older adults from diverse cultural contexts. This broader perspective provides valuable insights for early intervention programs and policies aimed at enhancing the lives of older adults (6, 7).

The novel findings of our study are crucial for understanding the various factors that contribute to QoL in older adults across different cultural settings, thus supporting their wellbeing and helping them age actively and healthily. By extending our analysis beyond specific health conditions and incorporating a wider range of geographical regions, we hope to inform the development of more inclusive and effective policies and interventions for older adults around the world. The World Health Organization (WHO) as part of its Aging and Life Course Program has developed the “Active aging: a policy framework” to address this problem (8). The framework intends to inform and guide discussion and formulation of action plans that foster healthy and active aging.

The concept of active aging was defined by the WHO as “the process of optimizing opportunities for health, participation, and security to enhance quality of life as people age” (9). Active aging emerges as a strategy to achieve QoL, permeated and influenced by six determinants: physical environment, health and social services, social environment, economic, personal, and behavioral determinants (10). This multidimensional definition implies that this concept intersects with others, such as productive aging, healthy aging, and successful aging (11–13).

Although active aging and QoL have some overlap, by definition, active aging is considered a dynamic process, whereas the QoL is a “state of being” (9). A study has noted that elements that compose the active aging index also relate to the elements that define life satisfaction/life happiness as measured for QoL (6). Furthermore, another study, using a sample from 27 European countries, examined QoL among older adults as a subset of active aging (7).

Within this broad framework of active aging and QoL, engaging in social activities, along with better physical health, financial condition, and security, are the essential aspects of QoL as defined by older adults themselves (14, 15). The concept of QoL is at times used conversely with active aging but is mainly considered as an outcome or the proxy measure of active aging (1, 7, 16–18).

Previous studies have reported the association of QoL with regard to diseases and clinical conditions among older adults (3–5), but none have investigated the association of active aging determinants with QoL. In this study, we aimed to fill the knowledge gap by investigating the association between active aging determinants and quality of life (QoL) among older adults. Our research stands out from previous studies that mainly focused on the association of QoL with diseases and clinical conditions among older adults (3–5). By examining the association between active aging determinants and QoL, our study offers a more comprehensive understanding of these factors and their role in promoting active aging and better QoL for older adults. Understanding the association of active aging determinants and QoL may help policymakers plan an early intervention or program to assist the future older adult in aging actively by optimizing their quality of life. Ultimately, this will help in the comprehensive support of the aging population in physical, mental, social, and financial wellbeing. Thus, this study aims to demonstrate the association of active aging with QoL, describing the need for more all-inclusive and broader measures designed to incorporate these unique factors influencing healthcare, health outcomes, longevity, and overall QoL in older age.

## Methodology

### Protocol and registration

This systematic review of the Preferred Reporting Items for Systematic Reviews and Meta-Analyses Protocols (PRISMA-P) guidelines (19) and the study protocol were registered in the International Prospective Register of Systematic Reviews (PROSPERO): CRD42020186740.

### Eligibility criteria

Only studies published in the English language were considered in this review. Studies were included based on a series of predefined inclusion and exclusion criteria as follows:

#### Inclusion and exclusion criteria

The study used the following inclusion criteria: (i) published original articles that assessed the association between active aging (AA) components and QoL domains; (ii) studies published between 1 January 2000 and 31 July 2020; (iii) having individuals aged 60 years or older as the study sample; and (iv) interventional, cross-sectional, and longitudinal study designs. For QoL assessment, we considered studies that used self-reported QoL questionnaires and wellbeing scales containing QoL or Health-Related Quality of Life (HRQoL) domains (life satisfaction, wellbeing, and self-rated health) and specific domains that include QoL or HRQoL (physical, cultural, social, psychological, mental, and spiritual domains). In addition, we included studies that utilized other relevant QoL assessment tools, such as CASP, SF EQ5D, and VAS, due to their established validity in evaluating active aging and QoL. We decided not to limit the study search to those that assess QoL using only generic instruments (WHOQoL-100 or SF-36). As a result, we also included key intervention and cohort studies that assessed the association between elements of AA and QoL domains.

### Search strategy

We searched for relevant articles from various electronic databases, including MEDLINE/PubMed, EMBASE and Cochrane via OVID and Open Gray, LILACS, and CINAHL. We used keywords for active aging (health, participation, and security) and the population of interest (geriatrics, older adults, elderly, aged people, and seniors), in combination with the keyword for QoL (quality of life). Keywords were combined using the Boolean operators “AND” and “OR”. All identified articles were screened independently by two reviewers (RM and SS) (Appendix 1).

### Data selection and collection process

**Figure 1:**
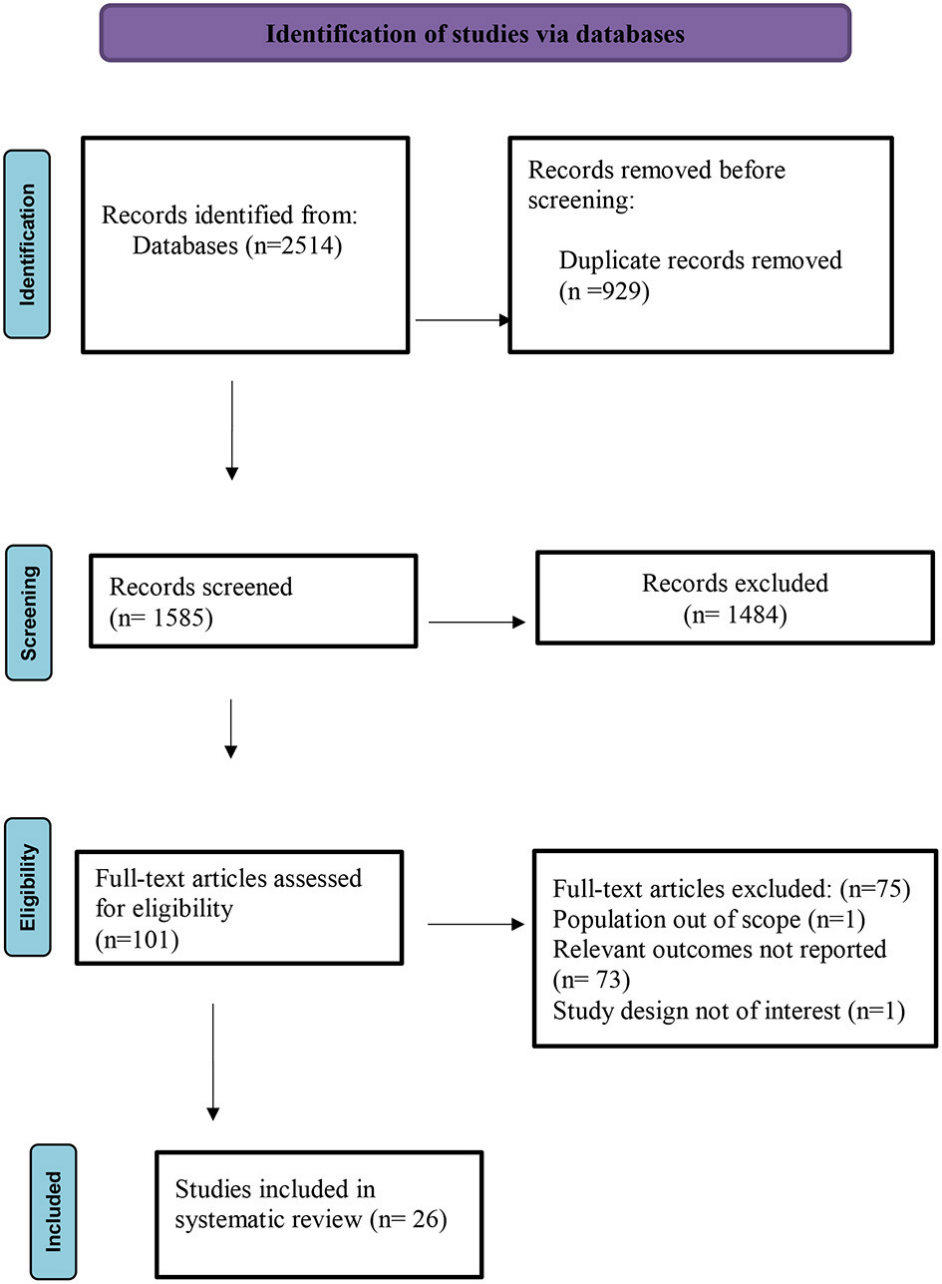
PRISMA flow chart for the inclusion and exclusion process.

The identified articles from the search were screened by two independent researchers/authors (RM and SS). At first, titles and abstracts of identified articles were assessed, and then eligible articles with full texts were retrieved and screened in full against the eligibility criteria mentioned above. All disagreements that arose were solved via discussion with a third reviewer (PKM, DM, or TTS). A flowchart detailing the study inclusion and exclusion process is included (Figure 1).

### Data extraction

Data were independently extracted by two reviewers (RM and SS) using a standardized data extraction template designed for this purpose. The following data and information were extracted from each of the included studies: country, study setting, sample type, and size, participants' age and gender, QoL measurement instruments (both generic and specific scales related to health, security, and participation), and active aging measures/definition. Disagreements at this stage were also solved via discussion with a third party/reviewer where necessary.

### Quality assessment

The quality of included studies was examined, independently, by two authors (RM and PK) using the Newcastle–Ottawa Quality Assessment Scale (NOS) of cross-sectional and cohort studies (20). Here, we determined the quality of selection, comparability, exposure, and outcome of each study participant, using a scoring system (maximum 9 points). The qualities of included studies were categorized into three: (1) high (score of 7–9); (2) moderate (score of 4–6); and (3) low (score of 0–3) qualities.

The Joanna Briggs Institute (JBI) Critical Appraisal Tool (21) was used to examine the methodological quality of interventional studies and the extent to which a study addressed the possibility of bias in its design, conduct, and analysis. The qualities of assessed studies were divided into three categories: (+) Yes implying low-risk bias; (?) unclear; and (–) No, implying high-risk bias. Disagreements were resolved through discussion to reach the final agreed score.

## Results

### Study selection process

Figure 1 presents the study selection process, which was divided into four key stages:

**(i) *Identification***: In July 2020, a database search was done through Central, Embase, Medline via OVID (2,502 articles), CINAHL (3 articles), LILAC, and Open Gray (5 articles), and bibliographic search of systematic literature reviews (SLRs) (4 articles). Thus, the initial search yielded 2,514 articles identified from the online databases. However, 929 were removed because they were duplicates.
**(ii) *Screening***: In total, 1,585 titles and abstracts were screened for eligibility. A total of 1,484 studies were removed because they did not meet the eligibility criteria such as population out of scope, intervention not of interest, relevant outcomes not reported, and study design and publication type not of interest.
**(iii) *Eligibility***: At this stage, 101 full-text articles were assessed. Of these, 75 studies were excluded after a full-text review because the population was out of scope, relevant outcomes were not reported, and study design was not of interest.
**(iv) *Included***: In total, 26 studies were considered to be eligible for inclusion in this systematic review.

### General characteristics of the studies

There were 22 cross-sectional, three longitudinal, and one quasi-experimental design studies—all studies composed exclusively of the older people (60 years or older) of both sexes (Table 2). Of the 26 studies, 14 studies were from seven Asian countries (China, India, Korea, Japan, Malaysia, Turkey, and Egypt). Two studies were conducted in the Latin American region (Brazil and Mexico) and four European regions (Austria, Ireland, UK, and Israel). One study each was conducted in Canada, Australia, and Nigeria.

Two contexts of the living arrangement were considered; community-based dependent older people and older people living in residential aged-care facilities. Eighteen studies included community-dwelling participants (22–26, 31, 32, 36–38, 41–48) and four studies included participants from residential aged care facilities (30, 34, 35, 39), while four studies did not report the kind of living arrangement (28, 29, 33, 40).

### Quality of studies

The qualities of cross-sectional and longitudinal studies were assessed through the NOS scale (Table 1). Based on the proposed cutoff points, 15 studies were classified as high-quality (22, 25, 28–31, 33, 38–42, 44–47) and 10 studies of medium quality (23, 24, 26, 28, 32, 35–37, 43, 48). The Joanna Briggs Institute (JBI) was used to evaluate the quality of the quasi-experimental study and scored 8/9 (88%) low risk of bias (34).

**Table 1:** Quality of studies assessed through the newcastle–ottawa quality assessment scale.

**References**	**Selection**				**Comparability**		**Outcome**			**Total quality score**
	**Representativeness of exposed cohort**	**Selection of non-exposed cohort**	**Ascertainment of exposure**	**Demonstration that outcome of interest was not present at the start of the study**	**Adjusted for the most important risk factors**	**Adjusted for other risk factors**	**Assessment of outcome**	**Follow-up length**	**Lost to follow-up rate**	
López-Ortega and Konigsberg (22)	1	1	1	1	1	0	1	1	0	7
Liu et al. (23)	1	1	1	1	0	0	1	1	0	6
Levasseur et al. (24)	1	1	1	1	0	0	1	1	0	6
Ramia and Voicu (7)	1	1	1	1	0	0	1	1	0	6
Puvill et al. (25)	1	1	1	1	1	1	1	1	0	8
Abdelbasset et al. (26)	1	1	1	1	0	0	1	1	0	6
Kim et al. (27)	1	1	1	1	1	0	1	1	0	7
Neri et al. (28)	1	1	1	1	1	0	0	1	0	6
Dahlberg and McKee (29)	1	1	1	1	1	1	0	1	0	7
Zhang et al. (30)	1	1	1	1	1	1	1	1	0	8
He et al. (31)	1	1	1	1	1	0	1	1	0	7
Ju et al. (32)	1	1	1	1	0	0	1	1	0	6
Choi et al. (33)	1	1	1	1	1	0	1	1	0	7
Onunkwor et al. (35)	1	1	1	1	0	0	1	0	0	5
Haider et al. (36)	1	1	1	1	0	0	1	1	0	6
Marques et al. (37)	1	1	1	1	0	0	1	1	0	6
Tavares et al. (38)	1	1	1	1	1	0	1	1	0	7
Top and Dikmetaş (39)	1	1	1	1	1	0	1	1	0	7
Park et al. (40)	1	1	1	1	1	0	1	1	0	7
Bilgili and Arpaci (41)	1	1	1	1	1	0	1	1	0	7
Sampaio et al. (42)	1	1	1	1	1	1	1	1	0	8
Layte et al. (43)	1	1	1	1	0	0	1	1	0	6
Sewo Sampaio and Ito (44)	1	1	1	1	1	1	1	1	0	8
Guedes et al. (45)	1	1	1	1	1	0	1	1	0	7
Gureje et al. (46)	1	1	1	1	1	0	1	1	0	7

Table 2 summarizes the instruments used to measure the QoL in the selected 26 studies. The concept of AA was measured, considering the three pillars of AA: participation, health, and security. The current study analyzed the active aging of the older population through their level of participation in physical, social, and cultural leisure activities about their socio-demographic characteristics and QoL dimensions in old age. In addition to participation, the health and security statuses have been also investigated in relation to QoL among older adults. The most widely used questionnaire to assess QoL is the World Health Organization Quality of Life Assessment–Module for Older Adults (WHOQoL-Old) (8 studies) (30, 36, 38, 39, 41, 42, 44, 45), followed by the WHOQOL–Abbreviated Version (WHOQoL-Bref) (6 studies) (35, 36, 42, 44, 46, 48) and the Short Form-36 (SF-36) (5 studies) (22, 23, 30, 31, 34). The European Quality of Life-5 Dimension (EQ-5D) (26, 40), Control, Autonomy, Self-Realization, and Pleasure (CASP-19) (28, 43), and visual analog scale (VAS) (32, 33) were used in two studies each. The following instruments were used in one study each: The Satisfaction with Life Scale (SWLS) (24), CASP-12 (25), SF-12 (47), WHO-5 (29), and CASP-16 (37).

**Table 2:** Summary study characteristics.

**References**	**Countries**	**Setting**	**Study design**	**Sample size (N)**	**Female %**	**QoL instruments**
López-Ortega and Konigsberg (22)	Mexico	Community-dwelling	Cross-sectional	295	43.5	SF-36
Liu et al. (23)	China	Community-dwelling	Cross-sectional	442	58.6	SF-36
Levasseur et al. (24)	Canada	Community-dwelling	Cross-sectional	155	60	SWLS
Ramia and Voicu (7)	India	Community-dwelling	Cross-sectional	160	100	WHOQOL-BREF
Puvill et al. (25)	Europe and Israel^*^	Community-dwelling	Cross-sectional	66,561	55.9	CASP-12
Abdelbasset et al. (26)	Egypt	Community-dwelling	Cross-sectional	184	29.9	EQ-5D
Kim et al. (27)	South Korea	Community-dwelling	Cross-sectional	517	89.2	SF-12
Neri et al. (28)	Brazil	Not reported	Longitudinal	7,651	53.2	CASP-19
Dahlberg and McKee (29)	UK	Not reported	Cross-sectional	1,255	61.8	WHO-5
Zhang et al. (30)	China	Residential aged care facility	Cross-sectional	1,369	60	WHOQOL-OLD, SF-36
He et al. (31)	China	Community-dwelling	Cross-sectional	2,644	59.19	SF-36
Ju et al. (32)	South Korea	Community-dwelling	Longitudinal	340	36.5	VAS
Choi et al. (33)	Korea	Not reported	Longitudinal	7,096	57.1	VAS
Rugbeer et al. (34)	Australia	Residential aged care facility	Quasi-experimental design	100	79	SF-36
Onunkwor et al. (35)	Malaysia	Residential aged care facility	Cross-sectional	203	32.5	WHOQOL-BREF
Haider et al. (36)	Austria	Community-dwelling	Cross-sectional	83	86	WHOQOL-BREF, WHOQOL-OLD
Marques et al. (37)	Brazil	Community-dwelling	Cross-sectional	1,197	64.5	CASP-16
Tavares et al. (38)	Brazil	Community-dwelling	Cross-sectional	1,691	63.7	WHOQOL-OLD
Top and Dikmetaş (39)	Turkey	Residential Aged care facility	Cross-sectional	120	36.66	WHOQOL-OLD
Park et al. (40)	South Korea	Not reported	Cross-sectional	229,226	2.719	EQ-5D
Bilgili and Arpaci (41)	Turkey	Community-dwelling	Cross-sectional	300	48.3	WHOQOL-OLD
Sampaio et al. (42)	Japan	Community-dwelling	Cross-sectional	465	NR	WHOQOL-BREF, WHOQOL-OLD
Layte et al. (43)	Ireland	Community-dwelling	Cross-sectional	6,279	NR	CASP-19
Sewo Sampaio and Ito (44)	Japan	Community-dwelling	Cross-sectional	465	48.6	WHOQOL-BREF, WHOQOL-OLD
Guedes et al. (45)	Brazil	Community-dwelling	Cross-sectional	1,204	53.57	WHOQOL-OLD
Gureje et al. (46)	Nigeria	Community-dwelling	Cross-sectional	2,152	NR	WHOQOL-BREF

NR, Not reported; ^*^27 European countries.

The selected studies in the present systematic review used different questionnaires for assessing active aging. We observed that the questionnaire assessed different determinants of active aging. For example, the WHOQoL–OLD and WHOQoL–BREF assessed the personal, social, behavioral, environment, health and social services, physical environment, and economic aspects of aging; while CASP-12, CASP-16, CASP-19, SF-12, SF-36, and EQ5D measured the three aspects of active aging, namely personal, behavioral, and social aspects. Similarly, VAS was used to assess the personal and behavioral aspects and SWLS and WHO-5 measured personal aspects only (Table 3).

**Table 3:** Similarities and differences of the questionnaires/instruments used.

	**Determinants of active aging**					
**Questionnaire**	**Personal**	**Behavioral**	**Social environment**	**Health and social services**	**Physical environment**	**Economic**
WHOQoL–OLD	√	√	√	√	√	√
WHOQoL–BREF	√	√	√	√	√	√
CASP-19	√	√	√			
CASP-16	√	√	√			
CASP-12	√	√	√			
SF-36	√	√	√			
SF-12	√	√	√			
EQ5D	√	√	√			
VAS	√	√				
SWLS	√					
WHO-5	√					

### Association of active aging determinants and QoL

Table 4 summarizes the key findings on the association between elements of AA and QoL domains. Various instruments were used to ascertain QoL scores, thus allowing a wide variety of QoL domains to be evaluated in the analyzed studies. The most examined QoL domains included physical health, mental health, functional capacity, psychological, emotional, social relationships, environment, pain, overall health, general QoL, and vitality concerning social participation and engagement in reading, art, and leisure activities.

**Table 4:** Association of active aging determinants and quality of life.

**References**	**Scale for QOL**	**QoL determinants**	**Results**
López-Ortega and Konigsberg (22)	SF-36	Personal factors, behavioral determinants, determinants of social environment	Measure: β-coefficient [95% CI] Physical functioning (*P < * 0.01), Vitality (*P < * 0.001) •Marital status: Widowed (compared to single and married) •Physical functioning *p* < 0.05, role limitations owing to physical-health problems *p* < 0.01, role limitations because of emotional problems *p* < 0.01, vitality *p* < 0.05, energy and fatigue, mental-health (psychological distress and emotional wellbeing) *p* < 0.05, social functioning *p* < 0.01, bodily pain *p* < 0.05 •Financial status: Poor (compared to good and fair financial status) •*p* < 0.05 in physical functioning, role limitations because of physical health problems, role limitations owing to emotional problems, vitality, energy and fatigue, mental health comprising of psychological distress and emotional wellbeing, social functioning, bodily pain, and *general health perception* •Concerning living arrangements and social support, the number of contacts with family members and close friends affected only physical function (*p < * 0.01), vitality (*p < * 0. 05), mental health (psychological distress and emotional wellbeing) *p* < 0.01, and social functioning *p* < 0.01 •Chronic diseases consistently had lower scores in all SF-36 dimensions, although only physical functioning (*p* < 0.05) and vitality (*p* < 0.01) had significant statistical differences •Household members living with the respondent and occupation did not affect the 8 domains of the SF-36. •Except for the general health domain (*p < * 0.01), the presence of chronic diseases did not affect estimated models on HRQoL domains.
Liu et al. (23)	SF-36	Personal factors, behavioral determinants, determinants of social environment	•The mental component summary (MCS) encircles the domains of Mental health, role limitations because of emotional problems, social functioning, as well as vitality. •In contrast, the physical component summary (PCS) encompasses a general perception of health, bodily pain, role limitations owing to physical problems, and physical functioning. •Older adults (married/widowed individuals) had significantly greater MCS and PCS scores compared to the never-married or divorced (*P* < 0.05). •Lower MCS scores were found among those who had < 5 h of sleep/day (*P* < 0.05) and those having a medical history of gastrointestinal disease (*P* < 0.001), urinary tract disease (*P* < 0.001), cancer (*P* < 0.05), or previous history of fractures (*P* < 0.001). •Hypertensive participants showed significantly lower PCS scores compared to non-hypertensive ones (*P* < 0.001). •Multivariable analysis results confirmed the descriptive comparisons, besides sleep time, which become non-significant. Unmarried or divorced participants had significantly lower PCS (*P* < 0.01) and MCS scores (*P* < 0.001). •History of chronic diseases such as gastrointestinal disease (*P* < 0.001), cancer (*P* < 0.05), urinary tract disease (*P* < 0.001), and previous history of fractures (*P* < 0.001) were associated with lower MCS scores. Only Hypertension was associated with lower PCS scores (*P* < 0.001). •No lifestyle factors (such as smoking) were associated with lower HRQoL on multivariable analysis.
Levasseur et al. (24)	The Satisfaction with Life Scale (SWLS)	Personal factors, behavioral determinants, determinants of social environment, determinants of physical environment	•There was no significant difference between the associations of SWLS with accomplishment level and satisfaction with social participation (Olkin's test: *P* = 0.71). In addition, the accomplishment level of social participation was not significant (*P* = 0.08) considering satisfaction with social participation (*P* = 0.02). •Younger age, no higher activity level, recent stressing event, level of activity perceived as stable, better wellbeing, and fewer obstacles in “Physical environment and accessibility” best explained higher social participation accomplishment level (*R*^2^ = 0.79; *P < * 0.001) •Apart from environmental factors, little variance (< 40%) was explained by each block in satisfaction with social participation compared to the accomplishment level of social participation. Better self-perceived health, level of activity perceived as stable, higher activity level, better wellbeing, and more facilitators in “Social support and attitudes” best explained greater satisfaction with social participation (*R*^2^ = 0.51; *P < * 0.001)
Puvill et al. (25)	CASP-12	Personal factors, behavioral determinants, determinants of social environment	•Approximately 0.17% and 0.33% of the variance in life satisfaction was attributed to ADL and IADL disability, respectively (both *p < * 0.001). •The impact of (I)ADL disabilities on life satisfaction was heaviest at age 50, which then decreased gradually with increasing age (*p*-trend < 0.001). Mental health accounted for more variance for depressive symptoms (5.75%) and loneliness (2.50%), but less variance for social resources (0.09% to 0.47%), all *p < * 0.001.
Ramia and Voicu (7)	WHQOL-BREF	Personal factors, behavioral determinants, determinants of social environment, determinants of health and social services, determinants of the physical environment, economic determinants	•The psychological domain had the least QOL score (mean ± SD: 36.7 ± 20), where more than 28% of older women had “very poor” QOL and 50.6% had moderately poor QOL. Physical- and health-related QOL had the highest mean score (49.5 ± 22), followed by environmental domain (47.38 ± 17) and social domain (43.7 ± 18), where 16.2, 16.2, and 14.4% of older women had “very poor” QOL, respectively. •Risk factors for poor QoL included absence of visits by friends and relatives (COR = 6.1, 95% CI: 1.69–21), age above 70 years (COR = 4.33, 95% CI: 2.21–8.48), neglecting attitude from family members (COR = 4.99, 2.44–10.19), and not having any role in family decisions (COR = 4.2, 95% CI: 1.83–9.56). In addition, low educational level, current and previous unemployment, and low personal and family monthly income were also risk factors, while living in urban areas was a protective factor. •Adjusted models showed age above 70 years (AOR = 11.3), non-possession of property (AOR = 9.0), neglecting attitude of family (AOR = 6.9), and absence of visit by friends and relatives AOR = 9.9) as risk factors, but urban residence, still, as a protective factor (AOR = 0.1) for poor QOL.
Abdelbasset et al. (26)	EQ-5D- VAS	Personal factors, behavioral determinants, determinants of social environment	•The psychological domain had the least QOL score (mean ± SD:36.7 ± 20), where more than 28% of older women had “very poor” QOL and 50.6% had moderately poor QOL. Physical- and health-related QOL had the highest mean score (49.5 ± 22), followed by environmental domain (47.38 ± 17) and social domain (43.7 ± 18), where 16.2, 16.2, and 14.4% of older women had “very poor” QOL, respectively. •Risk factors for poor QoL included absence of visits by friends and relatives (COR = 6.1, 95% CI: 1.69–21), age above 70 years (COR = 4.33, 95% CI: 2.21–8.48), neglecting attitude from family members (COR = 4.99, 2.44–10.19), and not having any role in family decisions (COR = 4.2, 95% CI: 1.83–9.56). In addition, low educational level, current and previous unemployment, and low personal and family monthly income were also risk factors, while living in urban areas was a protective factor. •Adjusted models showed that age above 70 years (AOR = 11.30, *P < * 0.001), non-possession of property (AOR = 9.0, *P < * 0.001), neglecting attitude by family members (AOR = 6.9, *P < * 0.001), and absence of visit by friends and relatives (AOR = 9.9, *P < * 0.001) were risk factors, while urban residence, still, a significant protective factor (AOR = 0.10, *P < * 0.001).
Dahlberg and McKee (29)	WHO-5 (wellbeing)	Personal factors, behavioral determinants, determinants of social environment, determinants of health and social services, determinants of physical environment, economic determinants	•Neighborhood exclusion accounted for more variance in wellbeing domain in rural compared to urban areas, while exclusion from services accounted for more variance in urban compared to rural areas. •Social exclusion domain: bivariate associations (**beta coefficient**) among social indicators Civic activity 1. Civic non-engagement: −0.09 2. Non-voting behavior: −0.12 3. Low competence for civic participation: −0.21 Material resources 1. Income discomfort: −0.20 2. Non-homeownership: −0.11 3. Low financial resources: −0.13 Social relations 1. Non-cohabitation: −0.08 2. Low contact with friends: −0.18 3. Low social resources: −0.34 Services 1. Poor access to care: −0.24 2. Poor access to amenities: −0.38 3. Poor public transport: −0.18 Neighborhood exclusion 1. Neighborhood alienation: −0.26 2. Neighborhood threat: 0.02 3. Neighborhood indifference: −0.12 There was no significant association between residence area (rural/urban) and age, gender, and years at the current address.
Neri et al. (28)	CASP-19	Personal factors, behavioral determinants, determinants of social environment	•Perceived QoL was associated with age, mobility, schooling, sociability, instrumental, and emotional support •Participation in social activities (proximal levels); No = 27.6%, Yes = 28.1%, and PR (95% CI): 1.07 (0.87–1.34) Participation in social activities (intermediate level); No = 28.2%, Yes = 27.9%, PR (95% CI): 1.06 (0.94–1.18) •Participation in social activities (distal level); No = 27.0%, Yes = 28.9%, PR (95% CI): 1.11 (1.01–1.22)
Kim et al. (27)	SF 12	Personal factors, behavioral determinants, determinants of social environment	•Lower PCS scores were associated with older age (*OR =* 0.97, 95%CI: 0.94–1.00), having more social support from significant others (*OR =* 0.88, 95% CI: 0.79–0.97), and having an income level of (300,000–390,000 KRW) (*OR =* 0.68, 95%CI: 0.47–0.99). •Good MCS scores were associated with living alone for over 20 years (*OR =* 0.63, 95%CI: 0.45–0.89), performing moderate physical activity (*OR =* 1.61, 95%CI: 1.08–2.38), and receiving social support from significant others (*OR =* 1.20, 95%CI: 1.08–1.34) and friends (*OR =* 1.19, 95%CI: 1.07–1.33). •On controlling for significant demographic variables, social support from significant others had a significant association with a lower PCS score (*OR =* 0.88, 95%CI: 0.79–0.98). However, social support from significant others (*OR =* 1.18, 95%CI: 1.05–1.33) and friends (*OR =* 1.16, 95%CI: 1.03–1.30) has a significant association with higher MCS scores.
Zhang et al. (30)	SF 36; WHQOL-OLD	Personal factors, behavioral determinants, determinants of social environment, determinants of health and social services, determinants of physical environment, economic determinants	•Regarding the physical component of the older participants' HRQOL, exercise, and labor-related factors accounted for the most change in the R^2^ value (0.116) •While concerning the mental component, sleep-related (0.054) and leisure-time-activity-related factors (0.053) accounted for the most change in the R^2^ value. •Regarding the older adults-specific HRQOL, the leisure-time-activity-related factors caused the biggest change in the R^2^ value (0.119), then exercise-and-labor-related factors (0.078).
He et al. (31)	SF-36	Personal factors, behavioral determinants, determinants of social environment	•Participating in social activities was associated with higher scores of health-related QoL. High educational level (OR = 1.59, 95%CI: 1.01–2.29), living alone or with a spouse (*OR =* 1.51, 95%CI: 1.08–2.12), high support utilization (*OR =* 1.13, 95%CI: 1.07–1.21), and high objective social support (*OR =* 1.08, 95%CI: 1.00–1.17) were associated with more social participation among older men. For the older women, high personal income (*OR =* 1.74, 95%CI: 1.25–2.43), single marital status (*OR =* 1.53, 95%CI: 1.11–2.10), overweight (*OR =* 2.28, 95%CI 1.24–4.19), normal weight (*OR =* 1.92, 95%CI: 1.10–3.34), living alone or with a spouse (*OR =* 1.55, 95%CI: 1.20–2.00), subjective (*OR =* 1.15, 95%CI: 1.10–1.20), and objective (*OR =* 1.11, 95%CI: 1.04–1.18) social support and were associated with more social participation.
Ju et al. (32)	VAS	Personal factors, behavioral determinants, determinants of social environment, economic determinants	•Participants who did not receive a national pension had a QoL of −4.40 (SE = 1.73; *P* = 0.0109), compared to those who had received one. •Moreover, those without a national pension and a low household income had the most significant decrease in QoL (−10.42; SE = 4.53; *P* = 0.0214). •Participants without national pensions and low wealth levels had a considerable decrease in QoL than those with a national pension and low wealth levels (−8.34; SE = 4.14; *P* = 0.0438).
Choi et al. (33)	VAS	Personal factors, behavioral determinants, determinants of social environment	•Individuals with changes from “participation to no participation” (b =2.25, *P < * 0.001), “no participation to participation” (b =3.35, *P < * 0.001), and “consistent participation” (b = 6.62, *P < * 0.001) were more likely to be satisfied with their lives compared to those with “consistent non-participation” (trend: *P < * 0.001). Furthermore, the impact of the positive relationship between consistent participation in social activity and quality of life changed across various aspects of social activity. •Religious activities, leisure/culture clubs, friendship organizations, family/school reunions, and voluntary work particularly had positive associations with consistent participation.
Rugbeer et al. (34)	SF 36	Personal factors, behavioral determinants, determinants of social environment	•A significant difference was found in social function post-training 2 times a week and 3 times a week. •Training three times a week showed an additional benefit in vitality. Improvements in the mental component summary scale post-training two times a week and three times a week were further noted.
Onunkwor et al. (35)	WHOQOL-BREF	Personal factors, behavioral determinants, determinants of social environment, determinants of health and social services, determinants of physical environment, economic determinants	•Gender had significant associations with all domains of QoL (*p*[[Inline Image]] < [[Inline Image]]0.05), and age was significantly associated with only the physical domain (*p* = 0.01). •The educational level had a significant association with the physical, psychological, and social domains (all *p*=0.01). Economic status had a significant association with the physical, psychological, and social domains (all *p*[[Inline Image]] < [[Inline Image]]0.05). •Duration of residence had a significant association with the psychological, social, and environment domains (all *p*=0.01). •Type of accommodation had a significant association with the psychological, social, and environment domains (all *p*[[Inline Image]] < 0.05). •Outdoor leisure activity, social support, and chronic co-morbidity had significant associations with all QoL domains (*p* ≤ 0.05). •Multivariable models showed that age, gender, economic status, outdoor leisure activity, chronic co-morbidities, and social support had a significant association with the physical QoL domain •The psychological domain had a significant association with gender, educational level, economic status, chronic co-morbidities, outdoor leisure activity, and social support. •The social domain had a significant association with gender, education level, duration of residence, outdoor leisure activity, chronic co-morbidities, and social support. Only chronic co-morbidities and social support had a significant association with the environment domain.
Haider et al. (36)	WHQOL-BREF	Personal factors, behavioral determinants, determinants of social environment	•Appendicular skeletal muscle mass (ASMM) had no role in the QoL context of prefrail and frail older adults, but balance and Daily Physical Activity had a role, as they had an association with social participation and autonomy. •Model 1: Daily physical activity, handgrip strength, and balance had significant associations with “overall QoL”. Balance was significantly associated with the QoL domains of physical health, psychological health, autonomy, environment, and social participation. Gait speed and chair stands were only associated with “social participation” only. •In model 2, independent variables explained overall QoL (R^2^ = 0.32), physical health (R^2^ = 0.20), autonomy (R^2^ = 0.247), and social participation (R^2^ = 0.356), and in which balance was the strongest determinant.
Marques et al. (37)	CASP-16	Personal factors, behavioral determinants, determinants of social environment	•Overall QoL mean score was 37.6% (95%CI: 37.2–38.1). •Older people with no probable cognitive deficit had higher QoL scores. •Individuals who remained living alone, continued to use the internet, began to work, and began to join groups also had higher QoL scores. QoL mean score of those who remained and became physically active was 41.5 and 40.1%, respectively. •Older adults who continued living with the family reduced QoL by 1.98 points (95% CI: −3.47; −0.50) compared to those who remained alone. However, those who started and remained working had higher QoL, 2.30 (95% CI: 0.45–4.16) and 3.90 (95% CI: 2.36, 5.44), respectively. •Regarding the internet, continued use was associated with a higher QoL score compared to those who stopped using it. All aspects of physical activity have a positive association with QoL scores compared to those who remained less active. •On multivariable analysis, older adults who remained living with their family had reduced QoL scores at 3.33 points (95% CI: −5.06; −1.60) compared to those who lived alone. Older adults who started to work had a positive QoL score (β = 2.82, 95% CI: 1.42–4.22). •Those who continued using the internet had 2.11 more QoL score points (95% CI: 0.85–3.36) compared to those who never used it. •Older adults who began participating in groups had higher QoL scores by 1.68 points (95% CI: 0.19–3.17) compared to those who did not participate. Regarding physical activity, all aspects remained with significant association. Older adults who remained physically active had higher QoL scores (β = 4.47, 95% CI: 3.32–5.63) than those who remained less active. Those who were sufficiently active in the first wave, but became less active, still had higher scores than those who remained less active. •Sensitivity analysis revealed associations only among older adults who moved with their family or a caregiver and those who remained working.
Tavares et al. (38)	WHQOL-BREF, WHQOL-OLD	Determinants of social environment	•The highest QoL mean scores were found in the social relationships domain (71.19) and topic of death and dying (74.30), while the environment domain (60.39) and topic of social participation (63.06) had the lowest scores. •The average score for self-esteem was (9.36 ± 4.09). •Lower self-esteem was associated with significantly lower QoL scores in all the WHOQOL-BREF domains and WHOQOL-OLD aspects (except death and dying) (*p* < 0.001).
Top and Dikmetaş (39)	WHQOL-OLD	Personal factors, behavioral determinants, determinants of social environment	•There was a significant association between QoL and attitudes to the aging of older adults. •The psychological growth subscale of attitudes to aging and sensory abilities subscale of QoL (r = 0.579, *P < *0.01) had the most significant relationship. Overall, QoL had a significant positive association with overall attitudes to aging (r = 0.408, *P < *0.01). •Dimensions of attitudes to aging (psychosocial loss, physical change, and psychological growth) were significant determinants of QoL among older adults. •Although gender did not affect overall QOL among older adults, happiness was a significant predictor of overall QOL.
Park et al. (40)	EQ-5D	Personal factors, behavioral determinants, determinants of social environment,	•Average QoL increased with the increasing amount of social activities individuals participated in (zero = 89.30, one = 93.28, two = 95.25, three = 96.27, four = 96.85). When individuals participated in one social activity, social activity had the strongest association with EQ-5D in the older adults age group regardless of gender. Moreover, more participation had a positive association with higher EQ-5D (p for trend < 0.0001). Among women, participating in relationship organizations was associated with a higher EQ-5D compared to participating in other types of social activities
Bilgili and Arpaci (41)	WHQOL-OLD	Personal factors, behavioral determinants, determinants of social environment, determinants of health and social services, determinants of physical environment, economic determinants	•Older men showed higher average scores for the sub-scales of sensory abilities, social participation, autonomy, past-present-and-future activities, and death-and-dying. However, older women showed higher average scores for the intimacy sub-scales and total average scores. Gender showed significant differences in the mean scores of sub-scales of autonomy, past-present-and-future activities, and intimacy (all *p < * 0.01). •Married older adults showed higher scores in the sub-scales of autonomy, social participation, past-present-and-future activities, and death-and-dying. However, unmarried older adults showed higher scores in the sub-scales of intimacy and sensory abilities. A significant difference in marital status showed significant differences in the mean scores of sub-scales of past-present and future activities, social participation, and death-and-dying (*t* = −2.00; (all *p < * 0.05). Married older adults had significantly higher total scores of QoL than the unmarried, (*p < * 0.05). •Older adults having a child showed higher scores in the sub-scales of sensory abilities and death-and-dying, while those without a child showed higher scores in other sub-scales. Furthermore, older adults with social security showed higher scores in the sub-scales of autonomy, social participation, past-today-and-future activities, and death-and-dying (*all p < * 0.01). Older adults having social security showed significantly higher total scores (*p < * 0.01). •Those with diseases showed significantly higher scores in the sensory abilities sub-scale than those without diseases (*p < * 0.01). However, older adults without the disease showed higher scores in the sub-scales of autonomy, social participation, and past-today and future activities (all *p < * 0.01). There was a statistically significant difference in the average total score of QoL according to disease state (*p < * 0.01). •Age (75 years and above) showed significant differences only in sensory abilities, social participation, and intimacy sub-scales (*all p < * 0.01). Those of 75 years and above had lower scores in social participation and intimacy sub-scales but higher scores in sensory abilities compared to those aged 60–65 and 66–74 years. •Educational levels of older adults showed significant differences in sensory abilities, autonomy, social participation, past-present and future activities, and death-and-dying (*all p < * 0.05) sub-scales (high school with higher scores, except in sensory abilities). •The person whom the older adults lived with showed significant differences in the QOL sub-scales of sensory abilities, past-today and future activities, death-and-dying, social participation, and intimacy (*all p < * 0.05).•Income level also showed significant differences in the sub-scales of autonomy, intimacy, past-today and future activities, death-and-dying, and social participation (*all p < * 0.01). Additionally, the total average score of the QOL sub-scales of the older adults was associated with their income (*p < * 0.01), with those in much/extreme financial difficulties having lower scores except for intimacy sub-scale scores •Correlation analysis showed a significant positive association of age with sensory abilities, but a negative association between age and social participation and intimacy scores (all *p < * 0.01). Additionally, the total average QoL score was positively associated with education but negatively associated with financial difficulties
Sampaio et al. (42)	WHQOL-OLD, WHQOL-BREF	Behavioral determinants, determinants of social environment	•Physical activity (β=0.21, *P < *0.01) had the highest influence on WHOQOL-BREF. This was followed by art activity (β = 0.17, *P < *0.01) and reading and writing (β=0.14, *P < *0.01). •Social activity (β=0.22, *P < *0.01) showed the highest influence on WHOQOL-OLD and then reading and writing activity (β = 0.12, *P < * 0.05).
Layte et al. (43)	CASP-19	Personal factors, behavioral determinants, determinants of social environment	•The average CASP-19 score was 43.8% (95% CI: 43.6–44.1) and was higher than the average score of the English Longitudinal Study of Aging, 42.5% (95%C1: 42.3–42.7). •Longevity was positively associated with high QoL provided it was accompanied by good levels of mental and physical health, social participation, and high-quality relationships. •Unadjusted analysis showed that CASP-19 was curvilinear with age, peaked at 67 years, and fell after that, while in the adjusted analysis, CASP-19 continued to rise, at a decreasing rate, with increasing age. •Variance in CASP-19 was largely attributed to mental health (7.6%) •Adjusting for variables in the mental health domain showed the lowest slope coefficient for the primary age term, which fell by 50% on adjusting for variables in all of the domains at once. There was a slight reduction in the positive slope coefficient of CASP-19 with age since the quadratic age term was significant and negative in all models
Sewo Sampaio and Ito (44)	WHQOL-OLD, WHQOL-BREF	Personal factors, behavioral determinants, determinants of social environment, determinants of physical environment	•Individuals living in urban areas showed higher total mean QoL scores compared to those in rural areas. •According to WHOQOL-BREF, those living in urban areas showed higher mean scores in the physical, psychological, and environmental domains (*all P < * 0.01) •Participants from urban areas also showed higher participation in reading and writing, contacts with distant friends and family, physical activities, and art activities compared to those from rural areas (*all P < * 0.01). However, those from rural areas were more engaged in work activities compared to their urban counterparts (*p < * 0.01). •There was a difference in essential activities in the occupational routine between urban and rural participants For the urban participants, the best model included work activity, physical activity, and reading and writing, while for the rural participants, art activity showed a relationship with QOL, other activities were not included.
Guedes et al. (45)	WHOQOL-OLD	Personal factors, determinants of social environment	•More physically active older adults (both genders) showed higher scores in the sensory ability, autonomy, and intimacy domains, as well as significantly higher overall QoL scores (all *P < * 0.05). •For women, those who were active and very active showed significantly higher scores in the social participation domain compared to sedentary women (*p* = 0.01), and the variation in scores also varied between genders. •Furthermore, significant differences were noted among men between those who were very active and sedentary, while among women, significant differences were noted between the active and the sedentary.
Gureje et al. (46)	WHQOL-BREF	Personal factors, behavioral determinants, determinants of social environment, economic determinants	•Economic status had a significant association with all four QoL domains. •Considering health variables, functional disability, and self-rated overall health were the most significant, while participation in community activities was the most significant social determinant

Six out of six studies using WHQOL-Bref showed that social participation and other activities such as reading, art, and physical activities significantly influence the QoL (35, 36, 42, 44, 46, 48). Seven out of eight studies using WHOQOL-Old demonstrated the consistent positive influence of activities including social participation, participating in art activities, reading, etc. on the QoL (30, 38, 39, 41, 42, 44, 45). Three out of five studies employing SF-36 showed that social or community participation is a relevant factor influencing the QoL (23, 30, 31). Two studies using CASP-19 showed that social participation and interaction significantly influence QoL (28, 43).

## Discussion

This systematic review synthesized evidence on the investigation of the association of active aging with QoL determinants among older adults. To date, most of the studies targeting QoL are focused mainly on clinical conditions, and thus the association of active aging determinants and QoL is uncharted. Our systematic review shows that different types of assessment tools have been used for the evaluation of QoL considering different components of AA, which varied with sex, settings, and study design, and resulted in a wide variation in association of QoL and active aging determinants. Due to the importance of AA, as it could interfere with personal as well as relatives' life, the understanding of determinants that affect AA is essential. This review has enhanced our knowledge of active aging in context to the quality of life that may prove crucial in understanding how the QoL can be maintained simultaneously with active living among older adults. In summary, our study supports the notion that the better the active aging determinants, the better the QoL among older adults.

Among the selected 26 studies in the current systematic review, QoL was assessed using different tools. We observed that the use of different QoL questionnaires resulted from the inclusion of different active aging determinants (Table 4) and therefore, variable determinants have been studied in different studies. For instance, some studies investigate the influence of personal determinants only, while some consider physical factors and some considered multiple factors such as physical activity, social participation, and mental health. Although this discrepancy among the investigated determinants is due to the use of variable questionnaires, the most commonly used questionnaire in various studies was the World Health Organization Quality of Life Assessment–Module for Older Adults (WHOQoL-Old) followed by WHOQOL–Abbreviated Version (WHOQoL-Bref) and the Short Form-36 (SF-36) (5 studies). Similarly, the European Quality of Life-5 Dimension (EQ-5D), Control, Autonomy, Self-Realization, and Pleasure (CASP-19), and visual analog scale (VAS) were used in two studies each. While the Satisfaction with Life Scale (SWLS), SF-12, CASP-12, CASP-16, and WHO-5 were used in one study each.

In examining the relation between active aging determinants and QoL, our study emphasizes that QoL is higher with the better status of active aging determinants, although some contrary findings are observed. It is noticed from many listed studies that social participation and other activities, including reading, art, and physical activities, have a positive impact on QoL (23, 28, 30, 31, 35, 36, 38, 39, 41–46, 48), despite different questionnaires such as WHOQOL-Old, CASP-19, and WHOQoL-Bref were used. Sampaio et al. (42) showed that social activity has the most significant impact on WHOQOL-Old, ensued by reading and writing (42). Similarly, our systematic review also showed that financial security and ensuring care positively influenced the QoL (48). In addition to that, Rugbeer et al. (34) demonstrated that mental and social benefits could be achieved regardless of exercise frequency (34).

The recent study by López-Ortega and Konigsberg (22) considered multiple outcome measures using an SF-36 questionnaire and reported the positive influence of socioeconomic and social, educational, and marital statuses on HRQoL (22), but there was no effect on HRQoL. In addition, there was no effect on HRQoL concerning the number of family members and those having chronic disease conditions. In contrast, another study conducted in the same year in the Shaanxi province of China reported the effect of a chronic condition on physical and mental HRQoL (23). On the contrary, we also acknowledge that not all the possible active aging determinants were associated with QoL among the selected studies. The study by Top and Dikmetaş (39) did not observe a significant association between gender and overall QoL (39). Notably, Onunkwor et al. (35), conducted a study on 203 older adults aged >60 years and failed to associate multiple factors such as pension, ethnicity, marital status, and smoking and alcohol status with any of the domains of QoL (35). Another study by Gureje et al. (46) also did not observe any association between gender, marital status, educational level, and residence and the physical domain of QoL (46). However, one of the studies showed no impact of the recipient of a national pension on QoL among middle-high and high household income levels and wealth (32). Considering most of the studies are based on associations, we support the concept that the higher the score in active aging determinants, the better QoL among older adults. We compared our findings to those of previous studies that investigated the relationship between active aging and QoL (49–52). Our results were consistent with these studies, supporting the notion that higher scores in active aging determinants lead to better QoL among older adults. This finding underlines the importance of promoting active aging to achieve improved QoL outcomes for older adults. Furthermore, a study by Ahmad Bahuri et al. (51) focused on active aging awareness and QoL among pre-elder Malaysian public employees, emphasizing the need to promote active aging in this population to ensure better QoL outcomes (51). Ooi and Ong (52) investigated active aging, psychological wellbeing, and QoL among older adults and pre-older adults Malaysians during movement control periods. Their findings suggest that even in challenging situations like movement control periods, promoting active aging can contribute to improved psychological wellbeing and overall QoL (52).

Our study, therefore, suggests that QoL among older adults is higher among individuals who are advancing well in different active aging components such as health, participation, and security. Our study compiling the previous studies suggests that there is a necessity to manage active aging determinants for the maintenance of QoL properly. Identifying other possible determinants and enhancing the methods to improve those determinants may help improve the QoL among older adults.

### Strength and limitations

The main strength of our review is that this is the first study collating information on the association between AA determinants and QoL. Furthermore, stringent search strategy was used in the current study to identify the relevant areas and thus strengthen our interpretation that physical, social, and health determinants are closely associated with QoL. However, as with most of the reviews, our study also has some limitations. Our search was limited to the English language; therefore, any studies published in other languages might have been omitted. Additionally, our literature screening time frame was limited to 2000–2020 as the active aging concept was developed from 2002 onward. Therefore, there might be the possibility of missing any articles that have been published before 2002. Our study's generalizability and interpretation may be affected by factors such as small sample sizes, geographically limited scope, unclear sampling schemes, and imbalanced gender distribution. We recommend future studies prioritize nationally representative studies, detailed sampling schemes, and balanced gender distributions to address these limitations.

## Conclusion

The maintenance of QoL in advancing age among older adults is necessary from individual to family, society, and healthcare perspectives. Thus, the elucidation of the related active aging determinants associated with an individual's QoL among older adults is paramount. QoL is multifaceted and is affected by several factors. Previous studies mainly highlighted QoL and clinical condition association; however, the specific aging determinants' association with QoL remains unknown. This review identified and systematically compiled the associated determinants of active aging and QoL. While relatively few studies have been identified, suggesting AA determinants, promising findings pointing to more extensive associations exist. To conclude, the findings from this study could help to further illuminate which AA determinants are essential in the maintenance of QoL. A future study could evaluate the cost necessity to improve the associated active aging determinants of QoL to improve/maintain the overall QoL in a better state.

## Author contributions

RM, TS, DM, and PM contributed to conception and design of the study. RM, PK, and SS organized the database. RM, TS, DM, PM, PK, and SS performed the statistical analysis and wrote sections of the manuscript. RM, TS, DM, PM, PK, and SS wrote sections of the manuscript. All authors contributed to manuscript revision, read, and approved the submitted version.
