# Supplementary material for: Determinants of active aging and quality of life among older adults: systematic review
Source: Front Public Health. 2023 Jun 26;11:1193789. doi: 10.3389/fpubh.2023.1193789 (PMC10330697; doi:10.3389/fpubh.2023.1193789)
Supplement: Supplementary file 1 [file Table_1.pdf]

Appendix 1: Database(s): **EBM Reviews - Cochrane Central Register of Controlled Trials** May 2020**, Embase** 1974 to 2020 June 05**, Ovid MEDLINE(R) and Epub Ahead of Print, In-Process & Other Non-Indexed Citations, Daily and Versions(R)** 1946 to June 05, 2020

Search Strategy:

| # | Searches | Results |
|---|---|---|
| 1 | (geriatrics or older adults or elderly or aged people or old age).ti,ab. | 847074 |
| 2 | active ageing.mp. | 600 |
| 3 | healthy ageing.mp. | 3714 |
| 4 | productive ageing.mp. | 24 |
| 5 | successful ageing.mp. | 834 |
| 6 | independent living.mp. | 15957 |
| 7 | participation.ti,ab. | 360606 |
| 8 | security.ti,ab. | 104485 |
| 9 | or/2-8 | 481135 |
| 10 | (quality of life or QoL).ti,ab. | 819118 |
| 11 | (health related quality of life or HRQoL or health-related quality of life).mp. | 128087 |
| 12 | (The World Health Organization Quality of Life or WHOQOL).mp. | 9688 |
| 13 | (Short Form 36 or SF-36 or SF-6D).mp. | 85875 |
| 14 | LEIPAD.mp. | 65 |
| 15 | (Older People's Quality of Life or OPQOL).mp. | 134 |
| 16 | or/10-15 | 849733 |
| 17 | 1 and 9 and 16 | 3406 |
| 18 | limit 17 to human [Limit not valid in CCTR; records were retained] | 3186 |
| 19 | limit 18 to english language | 2593 |
| 20 | limit 19 to yr="2000 -Current" | 2514 |
| 21 | remove duplicates from 20 | 1585 |
